# Antibody Repertoires in Humanized NOD-scid-IL2Rγ^null^ Mice and Human B Cells Reveals Human-Like Diversification and Tolerance Checkpoints in the Mouse

**DOI:** 10.1371/journal.pone.0035497

**Published:** 2012-04-27

**Authors:** Gregory C. Ippolito, Kam Hon Hoi, Sai T. Reddy, Sean M. Carroll, Xin Ge, Tobias Rogosch, Michael Zemlin, Leonard D. Shultz, Andrew D. Ellington, Carla L. VanDenBerg, George Georgiou

**Affiliations:** 1 Section of Molecular Genetics and Microbiology, University of Texas at Austin, Austin, Texas, United States of America; 2 Department of Biomedical Engineering, University of Texas at Austin, Austin, Texas, United States of America; 3 Department of Biosystems Science and Engineering, Eidgenössische Technische Hochschule Zurich, Zurich, Switzerland; 4 Department of Chemical Engineering, University of Texas at Austin, Austin, Texas, United States of America; 5 Chemical and Environmental Engineering, University of California, Riverside, California, United States of America; 6 Department of Pediatrics, Philips-University, Marburg, Germany; 7 The Jackson Laboratory, Bar Harbor, Maine, United States of America; 8 Institute for Cellular and Molecular Biology, University of Texas at Austin, Austin, Texas, United States of America; 9 Department of Pharmacology and Toxicology, University of Texas at Austin, Austin, Texas, United States of America; Children’s Hospital Boston/Harvard Medical School, United States of America

## Abstract

Immunodeficient mice reconstituted with human hematopoietic stem cells enable the *in vivo* study of human hematopoiesis. In particular, NOD-*scid*-*IL2Rγ^null^* engrafted mice have been shown to have reasonable levels of T and B cell repopulation and can mount T-cell dependent responses; however, antigen-specific B-cell responses in this model are generally poor. We explored whether developmental defects in the immunoglobulin gene repertoire might be partly responsible for the low level of antibody responses in this model. Roche 454 sequencing was used to obtain over 685,000 reads from cDNA encoding immunoglobulin heavy (IGH) and light (IGK and IGL) genes isolated from immature, naïve, or total splenic B cells in engrafted NOD-*scid*-*IL2Rγ^null^* mice, and compared with over 940,000 reads from peripheral B cells of two healthy volunteers. We find that while naïve B-cell repertoires in humanized mice are chiefly indistinguishable from those in human blood B cells, and display highly correlated patterns of immunoglobulin gene segment use, the complementarity-determining region H3 (CDR-H3) repertoires are nevertheless extremely diverse and are specific for each individual. Despite this diversity, preferential D_H_-J_H_ pairings repeatedly occur within the CDR-H3 interval that are strikingly similar across all repertoires examined, implying a genetic constraint imposed on repertoire generation. Moreover, CDR-H3 length, charged amino-acid content, and hydropathy are indistinguishable between humans and humanized mice, with no evidence of global autoimmune signatures. Importantly, however, a statistically greater usage of the inherently autoreactive IGHV4-34 and IGKV4-1 genes was observed in the newly formed immature B cells relative to naïve B or total splenic B cells in the humanized mice, a finding consistent with the deletion of autoreactive B cells in humans. Overall, our results provide evidence that key features of the primary repertoire are shaped by genetic factors intrinsic to human B cells and are principally unaltered by differences between mouse and human stromal microenvironments.

## Introduction

Humanized mouse models have become invaluable research tools for the study of *in vivo* human biological processes such as hematopoietic development [1], [2]. In these models, transplantation of stem cells into immunodeficient mice leads to *in vivo* reconstitution of human cells and tissues [3], [4]. Many different immunodeficient mouse strains and sources for human stem cells have been investigated and reconstitution has been characterized extensively in the non-obese diabetic-*scid*-*IL2Rγ^null^* (NOD-*scid*-*IL2Rγ^null^*) mouse strain which, when engrafted with umbilical cord blood (UCB) CD34^+^ hematopoietic progenitors (HSC), leads to the development of a fully populated human immune system, including human B-cell subsets [5], [6], [7].

Human HSC differentiate and develop into progenitor, precursor, and eventually mature B cells within the complex microenvironmental niches of the bone marrow (BM). BM stromal cells supply several essential components such as CXC-chemokine ligand 12, FLT3 ligand, IL-7, and stem cell factor that can be important for the functional development of B cells or the diversification of their primary antibody repertoire [8]. The sources of antibody repertoire diversity distil to four classes generally: (i) diversity present in the multiple germline immunoglobulin gene segments, (ii) combinatorial diversity of the recombining gene segments, (iii) junctional diversity caused by the imprecision of the recombinational joining process, and (iv) generation of somatic mutational diversity [9]. Antibody diversity within the primary B-cell repertoire is influenced by only the first three processes listed above. In addition to these genetic mechanisms, several developmental checkpoints against autoreactive B cells have been characterized among newly formed immature B cells in the BM and mature B cells in the peripheral blood of healthy humans whereby autoreactive B cells are stringently selected by clonal deletion, anergy, or receptor editing [10]. These checkpoints are oftentimes defective in patients with autoimmune diseases such as systemic lupus erythematosus (SLE) wherein a breakdown in early B-cell tolerance allows for the generation of self-reactive antibodies [11]. Furthermore, alterations in antibody diversity caused in part by the biased usage of particular V gene elements have also been described in SLE patients, including overrepresentation of the inherently autoreactive *IGHV4-34* heavy chain gene [12], [13] and the *IGKV4-1* light chain gene [11], even though consistent alterations of receptor editing have not been established.

Although B cells differentiate in immunodeficient mice engrafted with human hematopoietic stem cells, the sequence diversity of the humanized B-cell antibody repertoire has never been characterized in depth. Aspects of diversity in a humanized repertoire which might deviate significantly from the normal counterpart observed among human peripheral blood B cells could have implications for the relevance of human immune system mouse models. Various accounts have described functionality of the engrafted human immune system following immunization with model antigens or viruses [14], [15]. It is notable that engrafted NOD-*scid*-*IL2Rγ^null^* mice to date have shown only impaired adaptive immunity demonstrated by generally low serum antibody titers and almost undetectable antigen-specific IgG antibody responses [5], [16]. This weakened adaptive response can be explained in part by the fact that human T cells are selected based on murine MHC II (expressed on mouse thymic stromal cells) which might alter human T cell help. It is important, however, to determine other factors which might affect immune function. One open question is the extent to which an engrafted human immune system is similar, or dissimilar, to a human B-cell antibody repertoire. Therefore we initiated a high-resolution study coupling high-throughput deep sequencing with extensive bioinformatic analysis to compare the diversity of engrafted human B-cell repertoires in NOD-*scid*-*IL2Rγ^null^* mice and human peripheral blood B cells.

Using high-throughput sequencing, we obtained a combined total of >1,600,000 sequence reads from the mRNA/cDNA of three humanized mouse spleens and BM, and of peripheral blood mononuclear cells from two anonymous human donors. Our results demonstrate that humanized mice generate extensively diverse repertoires that display a highly similar pattern of V, D, and J family and individual segment usage in both V_H_ and V_L_ (Vλ and Vκ) genes to human peripheral B cells. However, two notable exceptions were observed: (i) A statistically significant elevation in the *IGHD7-27* gene, which is preferentially utilized during normal human fetal development and presumably reflects the umbilical cord origin of the engrafted CD34^+^ HSCs; (ii) The potentially autoimmune *IGHV4-34* and *IGKV4-1* gene segments were significantly elevated in newly-formed immature B cells relative to naïve or splenic B cells in humanized mice, a finding that bears striking analogies to the deletion of autoreactive V genes during hematopoiesis in the human [12], [13], [17], [18], [19]. Furthermore humanized mouse repertoires exhibited immunoglobulin (Ig) heavy chain CDR-H3 hallmarks, namely length, junctional diversity, hydropathy, and amino acid (a.a.) composition, devoid of classic autoimmune signatures such as the enrichment for positively charged residues or exceptionally long CDR-H3 sequences. Overall, our approach to repertoire profiling based upon bioinformatic analysis of high-throughput sequencing reveals that human HSC engrafted NOD-*scid*-*IL2Rγ^null^* mice develop diverse human Ig repertoires representative to those of peripheral blood B cells and suggests that, surprisingly, this animal model recapitulates some aspects of B-cell tolerance mechanisms observed in humans.

## Materials and Methods

### Ethics Statement

The animals used in this study were maintained under pathogen-free conditions in the barrier facility at the Animal Resource Center of the University of Texas at Austin. All experiments were conducted following the guidelines of the university’s Institutional Animal Care and Use Committee (protocol number AUP-2010-00089). This study is approved by the Institutional Biosafety Committee at the University of Texas at Austin (2010-06-0084).

### Mouse Strain, Husbandry, and Engraftment

NOD.Cg-*Prkdc^scid^ IL2rg^tm1Wjl^* (NOD-*scid*-*IL2Rγ^null^*) mice were developed at the Jackson Laboratory (Bar Harbor, ME). Breeding pairs were initiated with six-week-old female and eight-week-old male NOD-*scid*-*IL2Rγ^null^* mice.

NOD-*scid*-*IL2Rγ^null^* mice were irradiated with 100 cGy of gamma irradiation at 1-2 days of age. Intracardiac injection with 50 µL containing 3×10^4^ human CD34^+^ hematopoietic progenitors derived from UCB (Lonza, Cat. 2C-101B) was performed on irradiated pups. The engrafted mice (HuMs-1 and HuMs-2) were injected with UCB progenitors from a single donor; HuMs-3 was engrafted with UCB on a separate day using progenitors from a different donor.

### Immune Cell Isolation, Flow Cytometry, and Sorting of Humanized (HuMs) B Cells

At 16–18 weeks post-engraftment, spleen, femurs, and tibias of NOD-*scid*-*IL2Rγ^null^* mice (humanized mice) were harvested and single-cell suspensions were prepared in ice-cold sterile-filtered PBS with 0.1% BSA and 2 mM EDTA (buffer no.1). Human peripheral blood mononuclear cells (PBMCs) were collected from the whole blood of two anonymous, healthy females between 30–35 and 55–60 years of age (GCRBC, Galveston, TX). PBMCs were isolated using Histopaque-1077 solution (Sigma-Aldrich), washed twice, and resuspended in buffer no.1.

Fluorochrome-labeled antibodies were used according to the manufacturer’s instructions and HuMs cells were incubated for 15 minutes, washed, and resuspended in buffer no. 1 for immediate acquisition by flow cytometry. Mouse anti-human monoclonal antibodies (mAb) reactive with CD19 (clone HIB19), CD20 (2H7), CD38 (HIT2), CD27 (clone M-T271), CD24 (clone ML5), and CD45 (clone HI30) were purchased from BD Biosciences; goat anti-human IgM (catalog #2020) and IgD (catalog #2030) were acquired from Southern Biotechnology Associates. Analyses were performed on a FACSAria flow cytometer (BD) and analyzed using WEASEL or CellQuest analysis software. Isolation of naïve IgM^+^ IgD^+^ CD27^–^ B cells from humanized mouse spleens was performed using the Naïve B cell Isolation Kit II human (Miltenyi Biotec) per manufacturer’s protocol. The FACSAria was used to sort 55,000 immature B cells (CD24^hi^, CD38^+^ IgM^+^, and IgD^–^) from pooled humanized bone marrow (BM) directly into 1 mL of TRI reagent (Ambion).

### RNA Extraction and cDNA Generation

Total RNA was isolated from naïve or total B cells from humanized mouse spleens, pooled humanized mice immature B cells, or human PBMCs, and first-strand Oligo-dT cDNA was generated, followed by PCR to amplify the V_λ_, V_κ_, and V_H_ genes separately with a respective standard mix of optimized primers as shown in Table 1 [21]. Specifically, naïve or total B cells from humanized mouse spleens, pooled humanized mice immature B cells, or human PBMCs were lysed using the TRI reagent. Total RNA was isolated according to the manufacturer’s RiboPure Kit protocol (Ambion). First-strand cDNA generation was performed with 500 ng of isolated total RNA using SuperScript RT II kit (Invitrogen) and Oligo-dT primer. After cDNA construction, PCR amplification was performed to amplify the V_λ_, V_κ_, and V_H_ genes separately with a respective standard mix of primers described previously [20], [21]. PCR reactions were carried out as described previously [22] using the following conditions: 92°C denaturation for 3 min; 92°C 1 min, 50°C 1 min, 72°C 1 min for 4 cycles; 92°C 1 min, 55°C 1 min, 72°C 1 min for 4 cycles; 92°C 1 min, 63°C 1 min, 72°C 1 min for 20 cycles; and a final extension of 72°C for 7 minutes. PCR products were gel-purified before sequencing.

**Table 1:** PCR primers used in this study.

Primer Name	SEQUENCE (5' –>3')
**V_H_**	
VH1-fwd	CAGGTCCAGCTKGTRCAGTCTGG
VH157-fwd	CAGGTGCAGCTGGTGSARTCTGG
VH2-fwd	CAGRTCACCTTGAAGGAGTCTG
VH3-fwd	GAGGTGCAGCTGKTGGAGWCY
VH4-fwd	CAGGTGCAGCTGCAGGAGTCSG
VH4-DP63-fwd	CAGGTGCAGCTACAGCAGTGGG
VH6-fwd	CAGGTACAGCTGCAGCAGTCA
VH3N-fwd	TCAACACAACGGTTCCCAGTTA
IgM-rev	GGTTGGGGCGGATGCACTCC
IgG-all-rev	SGATGGGCCCTTGGTGGARGC
**Vκ**	
VK1-fwd	GACATCCRGDTGACCCAGTCTCC
VK246-fwd	GATATTGTGMTGACBCAGWCTCC
VK3-fwd	GAAATTGTRWTGACRCAGTCTCC
VK5-fwd	GAAACGACACTCACGCAGTCTC
VK1-rev	TTTGATTTCCACCTTGGTCC
VK2-rev	TTTGATCTCCASCTTGGTCC
VK3-rev	TTTGATATCCACTTTGGTCC
VK5-rev	TTTAATCTCCAGTCGTGTCC
**Vλ**	
VL1-fwd	CAGTCTGTSBTGACGCAGCCGCC
VL1459-fwd	CAGCCTGTGCTGACTCARYC
VL15910-fwd	CAGCCWGKGCTGACTCAGCCMCC
VL2-fwd	CAGTCTGYYCTGAYTCAGCCT
VL3-fwd	TCCTATGWGCTGACWCAGCCAA
VL3-DPL16-fwd	TCCTCTGAGCTGASTCAGGASCC
VL3-38-fwd	TCCTATGAGCTGAYRCAGCYACC
VL6-fwd	AATTTTATGCTGACTCAGCCCC
VL78-fwd	CAGDCTGTGGTGACYCAGGAGCC
VL1-rev	TAGGACGGTSASCTTGGTCC
VL7-rev	GAGGACGGTCAGCTGGGTGC

### High-throughput Sequencing of V_λ_, V_κ_, and V_H_ Repertoires and Bioinformatic Analysis

Roche GS-FLX 454 raw sequence reads (>1.6 million) were analyzed using the ImMunoGeneTics (IMGT) database [23] and IMGT/highV-QUEST program (version 1.0.3) to retrieve functional (productive) IGH, IGK and IGL transcripts. Results from IMGT were further parsed using Perl scripts for the compilation of relevant information for bioinformatic analysis, including the assignment of germline elements (V,D,J) and immunoglobulin isotype (IgM or IgG). Additionally we utilized a motif search script [22] in order to quantify several features of complementarity determining region 3 (CDR3) V_H_ and V_L_ sequences, namely: CDR3 frequencies, a.a. distributions, and hydropathy. We also employed an analysis package [24] to deconstruct V_H_ CDR3 (CDR-H3) junctional diversity caused by the imprecision of the recombinational joining process, as well as to examine the somatic mutation among full-length immunoglobulin transcripts. For the detection of autoimmune CDR-H3 motifs, a scoring scheme was used to determine autoreactive motifs such as polymeric stretches of Arginine (R) and/or Lysine (K) comprising the R/K rich regions. Raw sequence reads in sff format are deposited to Sequence Read Archive (SRA) at NCBI (Accession SRA049345.2).

### Statistical Methods

Population means for normally distributed data (CDR-H3 overall length and individual CDR-H3 components) were analyzed using an unpaired two-tailed Student’s *t* test. P values for gene repertoire analyses compared between any two unpaired samples, the frequencies of individual amino acids, and the analysis of positive charges in CDR-H3 were calculated using Chi-square test. Nonparametric Spearman correlation was used to quantify association in Ig gene family usage across all samples.

## Results

### B Cell Isolation from Human CD34^+^ Stem-cell Engrafted NOD-*scid*-*IL2Rγ^null^* (humanized) Mice

**Figure 1:**
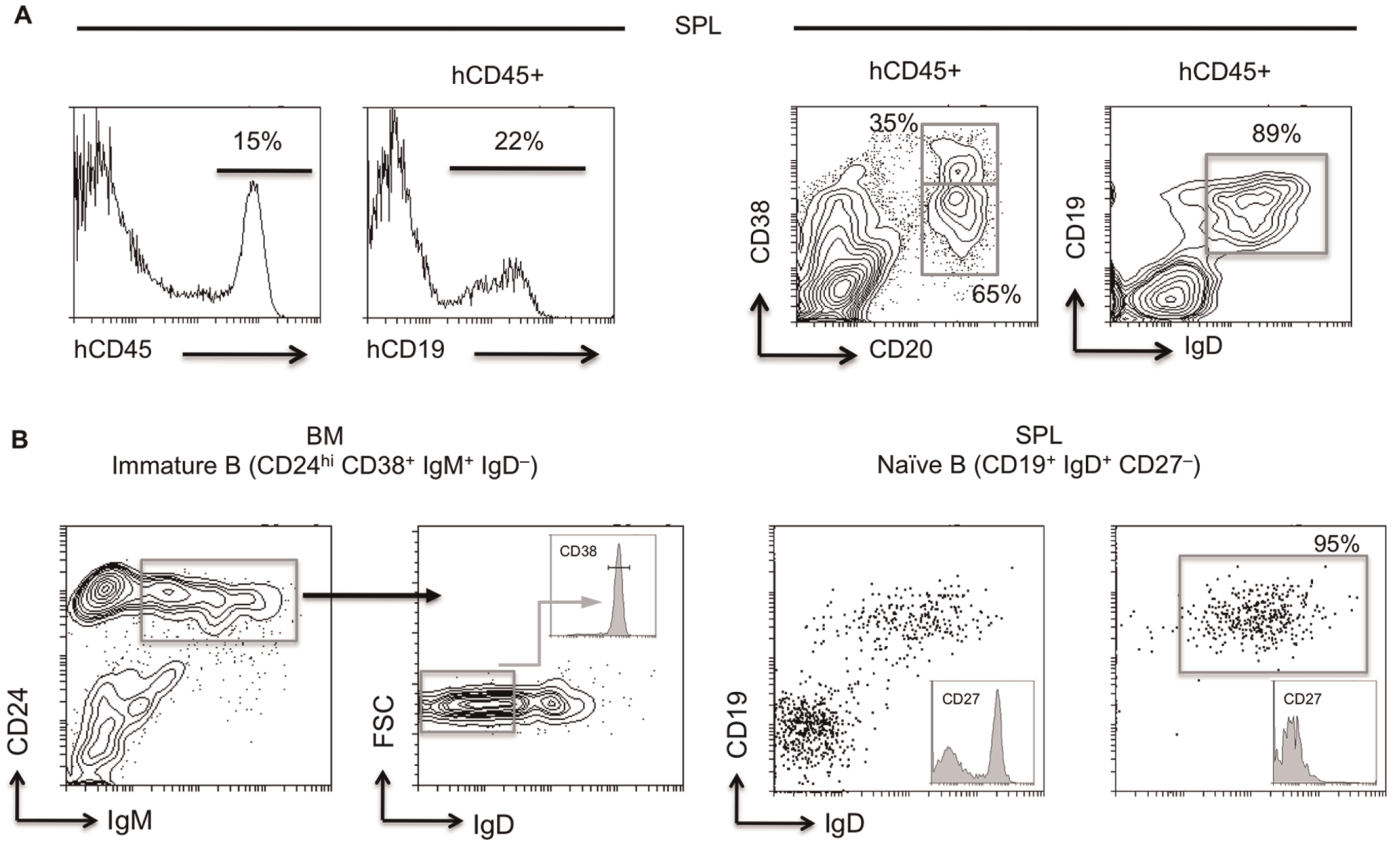
Human B lineage cells generated in humanized NOD-*scid-IL2Rγ^null^* mice. NOD-scid-IL2Rγ^null^ mice were engrafted with CD34^+^ stem cells obtained from human umbilical cord blood. Cells were recovered from bone marrow and spleen 16–18 weeks post-engraftment and examined by flow cytometry. (A) Total mononuclear cells isolated from the spleen (SPL) of a humanized NOD-scid-IL2Rγ^null^ mouse show the presence of 15% human CD45^+^ leukocytes in which CD19^+^ B lymphocytes comprise nearly one-fourth of the compartment. B cells were predominantly CD20^+^ CD38^int/lo^ and were chiefly IgD+ naïve cells (>90% on average in all three humanized mice). Results shown are representative of n = 3 humanized mice. (B) Figures depict the schemes used for sorting newly formed immature B cells (CD24^high^ CD38^+^ IgM^+^ IgD^–^) in bone marrow (BM), and naïve B cells (CD19^+^ IgD^+^ CD27^–^) in spleen. Boxes indicate the B cell compartments that were sorted for immunoglobulin gene sequencing.

In two independent experiments NOD-*scid*-*IL2Rγ^null^* mice were engrafted with UCB CD34^+^ hematopoietic progenitors and analyzed at either 16 or 18 weeks of age. CD19^+^ B cells comprised approximately 25% of CD45^+^ human leukocytes in the spleen and ∼90% co-expressed IgM/IgD while negative for the memory cell marker CD27 (Figure 1). The predominance of naïve IgM^+^IgD^+^ B cells in the periphery of humanized mice was anticipated [14], [15], [25], [26]. Naïve B cells (CD19^+^IgD^+^CD27^–^) were enriched from two humanized mice (HuMs-1NSpl and HuMs-2NSpl) by magnetic cell separation to an average purity of ≥95% (Figure 1). Total spleen cells were isolated from one additional humanized mouse (HuMs-3TSpl). Finally, 55,000 newly formed, immature B cells (CD24^high^CD38^+^IgM^+^IgD^–^) were sorted by flow cytometry from the pooled BM of the three animals, HuMs –1, –2, –3 (HuMs-ImmB) (Figure 1).

### V Gene Library Construction, Roche 454 DNA Sequencing and Bioinformatics Analysis

B cell populations were used to generate total cDNA, and human VH, Vλ, and Vκ genes were amplified using a well characterized and extensively validated set of 5′ oligonucleotide primers and a second set of five 3′ primers complementary to the constant regions of IgM, IgG1, IgG2, IgG3 and IgG4 [20], [21]. To place VDJ usage, CDR3 composition and somatic hypermutation (SHM) rates in the HuMs B-cell populations in context, we also amplified V genes from peripheral blood mononuclear cells (PBMCs) from two healthy adult human volunteers (HuPBC-1, HuPBC-2). Even though the cost per base for Roche 454 sequencing technology is >30 fold higher compared to the Illumina HiSeq platform employed in recent human immune repertoire studies [27], we elected to use the former because of its unique ability to provide reads of >380 bases that cover the entire IgH variable region. Large numbers of long reads are critical for delineating subtle features of Ig gene repertoires, the determination of SHM rates, and for Ig transcript abundance analyses (see below). Approximately 390,000 and 270,000 VH reads and between 26,000–150,000 Vκ and Vλ reads were obtained for HuPBC-1 and HuPBC-2 (Table 2). The number of reads for each of the humanized mice varied between 69,000–135,000 for VH, 23,000–73,000 for Vκ and 15,000–48,000 for Vλ. After an initial pass through the IMGT/highV-QUEST server [23] to identify functional (productive) Ig joins, a hidden Markov model motif-search [22] was used to first identify the CDR3 region in V_H_ and V_L_ genes. CDR3 frequencies, a.a. distributions including numbers of positively charged amino acids and hydropathy were calculated. Another analysis package [24] was used to deconstruct CDR-H3 junctional diversity caused by the imprecision of the recombinational joining process, as well as to examine the degree of SHM among full-length Ig transcripts.

**Table 2:** Summary of sequence reads generated for each humanized mouse (HuMs), pooled humanized mouse immature B cells (HuMs-ImmB), and human peripheral blood B cell samples (HuPBC).

V_H_	Total reads	Reads with identified germlines (IMGT)	Functional Sequences	Unique Sequences	IgM/IgG/Unidentified (%)
HuMs-1NSpl	134510	88879	51092	32520	96.43/0.36/3.21
HuMs-2NSpl	92710	62826	34111	27448	92.23/0.53/7.15
HuMs-3TSpl	69694	44687	24212	17942	92.95/2.04/5.01
HuMs-ImmB	79654	52236	28581	16874	97.07/0.80/2.13
HuPBC-1	394237	372986	154354	90473	69.53/24.98/5.49
HuPBC-2	272439	258289	53732	44576	53.96/30.82/15.22
**Vκ**					
HuMs-1NSpl	49224	39470	32322	–-	–-
HuMs-2NSpl	61736	48578	39811	–-	–-
HuMs-3TSpl	22446	18075	15062	–-	–-
HuMs-ImmB	73461	60733	49167	–-	–-
HuPBC-1	46768	44962	26511	–-	–-
HuPBC-2	150060	93609	55485	–-	–-
**Vλ**					
HuMs-1NSpl	24622	11623	9749	–-	–-
HuMs-2NSpl	16412	7780	6724	–-	–-
HuMs-3TSpl	15899	7705	6497	–-	–-
HuMs-ImmB	46608	21433	17871	–-	–-
HuPBC-1	26813	18103	7754	–-	–-
HuPBC-2	49945	6831	2533	–-	–-

As noted recently for cDNA-based B-cell repertoire profiling [28] and as shown in Table 2, there is a high rate of attrition of raw sequence reads from 454 (∼80%) as the data is filtered for full-length functional sequences and, ultimately, for unique sequences. We too observed Ig rearrangements with identical CDR3 amino acid sequence and the same component VDJ usage represented in multiple transcripts. Thus, when a molecular clonal expansion was identified in the 454 data set, only the longest representative sequence was chosen for estimating VDJ usage and repertoire features. It is not possible to ascertain whether identical transcripts reflect the presence of *bona fide* identical clones derived from clonal expansion, higher transcription levels in antibody secreting cells, or biases in cDNA generation during reverse transcription or PCR amplification. Nonetheless, earlier data [22] support the notion that large counts of identical transcripts (n>30) reveal the identity of highly transcribed V genes resulting from clonal expansion or differentiation to plasmablasts.

### Frequency Analysis Demonstrates Similar Polarization and Uniquely Diverse, Non-overlapping CDR-H3 Repertoires in Humanized Mice and Human Peripheral Blood B Cells

HuMs and HuPBC yielded 94,784 and 135,049 unique CDR-H3 sequences, respectively (Table 2). HuPBC repertoires were rather diverse as the top 20 CDR-H3 sequences (as defined by “clonal” transcript abundance) each accounted for only ∼0.10–0.5% of the total V gene cDNA counts. HuMs demonstrated an analogous degree of polarization; for example, the top 20 CDR-H3 frequencies in HuMs-1NSpl were in the range of ∼0.1–0.7%. In HuMs-ImmB the diversity was the greatest, as CDR-H3 frequencies in the top 20 were merely ∼0.1–0.2% and collectively accounted for only 2.1% of the entire repertoire. In both HuPBCs, the high frequency clones were predominantly composed of IgG. In HuMs samples the fraction of class switched Ig sequences in the repertoire was very low and hence all abundant CDR-H3s were IgM-derived. There was very little overlap among different individuals: the overlap of unique CDR-H3 sequences shared between any two HuMs repertoires was 0.02%; between the two HuPBC repertoires, 0.004%; and between any HuMs compared with HuPBC repertoire, 0%. These results indicate that both HuMs and HuPBC repertoires comprise highly diverse CDR-H3 a.a. sequences exhibiting little overlap or redundancy.

### Globally Similar Diversity of Ig Germline Family Usage in Humanized Mice and Human Peripheral Blood B Cells

**Figure 2:**
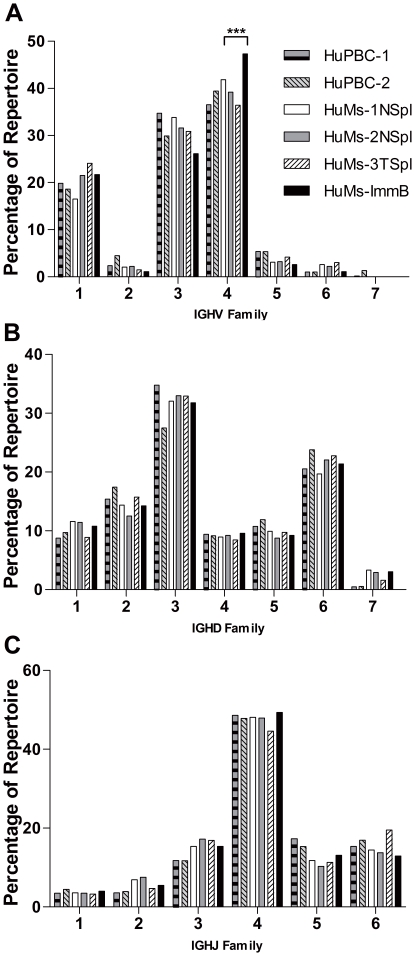
Human B cells generated in NOD-*scid*-*IL2Rγ^null^* mice produce a globally diverse V_H_ immunoglobulin repertoire that is indistinguishable from normal human peripheral blood B cells. B lymphocytes were obtained for high-throughput 454 GS-FLX immunoglobulin cDNA gene sequencing. The samples included peripheral blood B cells from two human donors (HuPBC-1, HuPBC-2), naïve B cells (CD19^+^IgD^+^CD27^–^) enriched from two humanized mouse spleens (HuMs-1NSpl and HuMs-2NSpl), total splenic B cells from one humanized mouse (HuMs-3TSpl), and newly formed immature B cells (CD24^high^CD38^+^IgM^+^IgD^–^) pooled from the bone marrow of all three mice (HuMs-ImmB). (A) Engrafted HuMs cells use the full range of IGHV families and display a pattern of utilization similar to control HuPBC samples. Frequencies of IGHV family member use by B cells arising in HuPBC, HuMs, and HuMs-ImmB are comparable between any two samples examined across all IGHV gene families (*r_s_≥*0.89 for all comparisons). One intrafamily statistical difference was discerned, however, for the IGHV4 family (^***^ *P*<0.0001; χ^2^). (B) Frequencies of IGHD family use are highly similar between HuMs and HuPBC (*r_s_≥*0.86 for all comparisons), although a subtle yet significant trend in elevated IGHD7 family use was discerned among HuMs samples (see text). (C) Frequencies of IGHJ use, like IGHV and IGHD, are statistically similar (*r_s_≥*0.71).

For IGHV family utilization in PBMCs an IgM-only analysis yielded quantitatively comparable results (data not shown). Therefore both IgM and IgG sequences were used for the VDJ frequency analyses presented below. Spearman correlation analysis of IGHV family member use by B cells arising in HuPBC, HuMs-Spl, and HuMs-ImmB showed comparable frequencies (*r_s_≥*0.89) between any two samples examined across all IGHV gene families. It was found that the pattern of IGHV family usage was conserved between HuPBC and HuMs samples, as IGHV families 1, 3, and 4 were highly over-expressed relative to IGHV 2, 5, 6, and 7 (Figure 2A). This trend of IGHV family usage corresponds approximately to previously published reports on repertoire bias in humans and generally parallels the genomic complexity of each IGHV family [29], [30], [31]. Individual V_H_ gene utilization between HuMs naïve B cells or total splenocytes and HuPBC repertoires was also conserved (Figure S1) as determined by Spearman correlation analysis (*r_s_* = 0.78). For example, the IGHV3 family members *IGHV3-23* and *IGHV3-30* were frequently utilized, representing a similar average of 9.1% (HuMs-1,2,3) and 10.1% (HuPBC-1,2) of total peripheral repertoires. Collectively, *IGHV4-34* plus *IGHV4-39* and *IGHV4-59* were the three most frequently arranged IGHV4 family members, representing a similar average of 26.5% (HuMs-1,2,3) and 28.3% (HuPBC-1,2) of all peripheral B-cell repertoires. However, one notable exception was observed: as discussed below, *IGHV4-34* was specifically and significantly enriched in the BM compartment of newly formed HuMs-ImmB cells relative to HuMs naïve B or total splenocytes (Figure S1). Lastly, it is of note that even in a data set comprising over 600,000 reads with identified IGHV genes from human PBMCs there was no evidence for usage of IGHV orphons or pseudogenes.

Similar to the global patterns of IGHV utilization, Spearman correlation analysis of IGHD and IGHJ gene families revealed no statistical differences between HuMs and HuPBC samples when examined across all families (Figure 2, B and C) (*r_s_≥*0.86). One particular intra-family difference, however, was observed within the single-member IGHD7 family, wherein the *D7-27* element, which is preferentially expressed during human fetal gestation [32], was used ∼10-fold more often in HuMs samples (∼3.0% of total) compared to HuPBC (∼0.3%) (*P* <0.0001; χ^2^). This aspect of the humanized repertoire may reflect the presence of both fetal and adult stem cells in the cord blood used for engraftment. Although the high degree of sequencing depth uncovered this fetal-like difference at the edges of the data set, a true fetal repertoire would be expected to have a greater *IGHD7-27* usage [32] and since the contribution of this gene segment was such a small proportion of the total HuMs repertoire (∼3.0%), we conclude that the HuMs humanized IGHD antibody repertoire is otherwise analogous overall to the repertoire of adult peripheral blood B cells.

**Figure 3:**
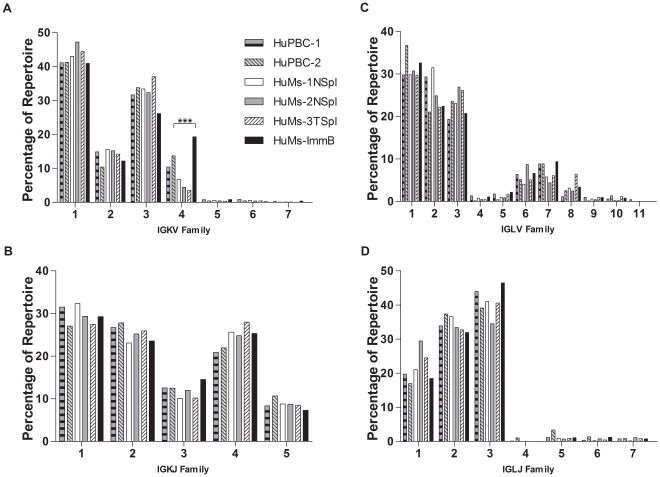
Human B cells generated in NOD-*scid*-*IL2Rγ^null^* mice produce a globally diverse V_L_ immunoglobulin repertoire that is highly similar to normal human peripheral blood B cells. Engrafted HuMs cells use the full range of IGHK and IGHL families and with a pattern of utilization similar to control HuPBC samples. Frequencies of IGHK and IGHL family member use by B cells arising in HuPBC, HuMs, and HuMs-ImmB are comparable between any two samples examined across all gene families: *r_s_≥*0.86 for *IGKV* (A); *r_s_≥*0.7 for IGKJ (B); *r_s_≥*0.85 for IGLV (C); *r_s_≥*0.75 for IGLJ (D). Although highly similar overall, an overrepresentation of the IGKV4 family occurred among HuMs-ImmB sequences when compared to HuPBC-2 or any other sample (***P<0.0001; χ^2^).

*IGKV* family usage patterns were conserved between naïve HuMs and HuPBC repertoires, with high expression of families 1 and 3, intermediate expression of 2 and 4, and very low expression of 5, 6, and 7 (Figure 3) with *r_s_* values *≥*0.86. Of note, however, *IGKV1-43*, which was present at a low frequency in HuMs samples, was not detected in the two HuPBC sets. We also noticed that IGKV4 family representation in immature B cells derived from HuMs BM was dramatically greater than in naïve or total splenocytes from HuMs or in human PBMCs (Figure 3A; Figure S2). Conservation of usage was observed in IGKJ for all 5 gene segments which were represented at relatively similar levels in all HuMs and HuPBC data sets (*r_s_≥*0.7, Figure 3B). Furthermore, we found no evidence of skewed downstream Jκ usage suggestive of light chain receptor editing among IGK genes from HuMs-ImmB cells [33]. In both total splenocytes and in naïve and immature HuMs B cells, as well as in HuPBC, IGLV usage was dominated by families 1, 2, and 3 (∼20–30%) with intermediate expression of 6 and 7 (∼10%) and very low expression of 4, 5, 8–11 (<5%, Figure 3C) (*r_s_≥*0.85, Figure S3).

### Peripheral Repertoires in Humanized Mice Display a Selective Loss of Autoreactive V Genes

**Figure 4:**
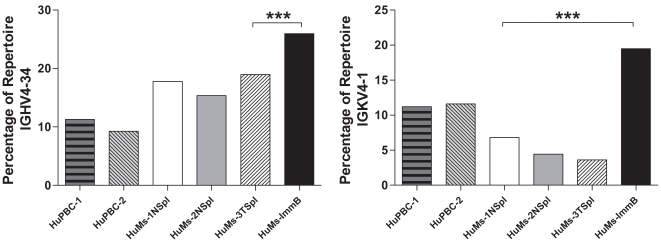
Deletion of autoreactive V genes during transit from bone marrow to the periphery. (A) Immature bone marrow B cells (HuMs-ImmB) were highly enriched for use of the inherently autoreactive *IGHV4-34* element compared to HuMs peripheral B cells obtained from sorted spleen naïve cells (HuMs-1NSpl, HuMs-2NSpl) or from total spleen cells (HuMs-3TSpl) (^***^ *P*<0.0001; χ^2^). (B) HuMs-ImmB were also highly enriched for utilization of the SLE-associated *IGKV4-1* light chain gene compared to HuMs peripheral B cells which had been depleted for this gene (^***^ *P*<0.0001; χ^2^).

*IGHV4-34* has been characterized as an “inherently autoreactive” IGHV gene [12], [34], which is strongly correlated with autoimmune diseases such as SLE [13] and hyper-IgM syndrome [19], which invariably encodes self-reactivity with carbohydrate antigens displayed at high density on erythrocytes and other cell types [35], [36]. In healthy humans B-cell tolerance occurs primarily at two checkpoints wherein developing B cells expressing highly polyreactive or autoreactive Ig receptors are deleted as the cells differentiate from early precursors to the immature stage in bone marrow to the mature naïve B-cell stage in the periphery [10], [17]. In striking analogy to the human checkpoint, *IGHV4-34* was significantly more highly represented in the compartment of newly formed HuMs-ImmB cells originating in the BM (25.9%; *P*<0.0001; χ^2^) but was subsequently depleted in maturing peripheral HuMs cells derived from either the naïve B-cell subset of spleen (HuMs-1,2; average 16.8%) or from total unfractionated spleen (HuMs-3; 19.5%) (*P*<0.0001; χ^2^) (Figure 4). Likewise, the *IGKV4-1* light chain gene has also been implicated in SLE and defects in B-cell tolerance [11], [18], [34] and a selective 3-fold loss of *IGKV4-1* in peripheral B cells (HuMs-1 and -2 NSpl and HuMs-3 TSpl) relative to HuMs-ImmB BM cells (*P*<0.0001; χ^2^) was detected (Figure 4).

### CDR-H3 Features of Length, Junctional Diversity Hydropathy, and a.a. Usage in Humanized Mice is Indicative of Normal B-cell Diversification and Tolerance

**Figure 5:**
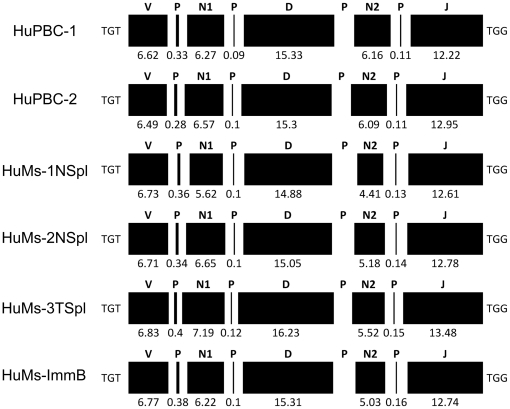
DNA deconstruction reveals IgM CDR-H3 repertoires that are normally diversified and indistinguishable between humanized NOD-*scid*-*IL2Rγ^null^* mice and normal human B cells. The germline contribution of IGHV, IGHD, and IGHJ elements is illustrated. Shown are IgM CDR-H3 sequences containing an identifiable IGHD segment located between the IGHV conserved cysteine codon (TGT) and the conserved tryptophan codon (TGG) encoded by IGHJ elements. All components are shown to scale. Data comprises functional IgM sequences derived from unique CDR-H3s isolated from HuPBC-1, HuPBC-2, HuMs-1NSpl, HuMs-2NSpl, HuMs-3TSpl, and HuMs-ImmB using a previously published algorithm [24]. Preservation of IGHV, IGHD, and IGHJ germline sequences were statistically similar across all samples (Student’s *t* test). The degree of nontemplated N-region [N] additions, palindromic [P] nucleotide additions, and total CDR-H3 length were also statistically indistinguishable, except for a subtle increase in 3′ N-region addition among HuPBC (*P* = 0.03).

As a means to characterize primary CDR-H3 repertoires, we focused exclusively on IgM transcripts. Whereas the HuMs Ig repertoires were predominantly of IgM isotype not only in BM immature and naïve splenocyte B cells but also in the total whole splenocytes (TSpl), the HuPBC repertoires were heavily populated by IgG class-switched transcripts. IgG transcripts were excluded from this analysis since they derive from secondary events such as clonal expansion and affinity maturation. CDR-H3 lengths of IgM genes were normally distributed for the two HuPBC samples, with an average length of 15.54 and 15.83 a.a.s. The HuMs samples were alike in length at an average of 15.57 a.a.s for HuMs-1 & -2 naïve cells, 16.52 a.a.s for HuMs-3 isolated from whole spleen, and 15.42 a.a.s for HuMs-ImmB cells. As a complement to CDR-H3 length analysis, we also deconstructed nearly 200,000 IgM CDR-H3 intervals to examine the global composition of junctional diversity. The germline preservation or the exonucleolytic loss of recombining germline elements, coupled with nontemplated N-region (N) and palindromic (P) nucleotide additions, created CDR-H3 repertoires that were statistically indistinguishable between HuMs and HuPBC repertoires in all but one regard (3′ N-region addition was subtly increased in HuPBC; *P* = 0.03) (Figure 5). We interpret this result as strong evidence for conserved genetic mechanisms of VDJ recombination that produce *bona fide* human B-cell repertoires in humanized NOD-*scid*-*IL2Rγ^null^* mice.

**Figure 6:**
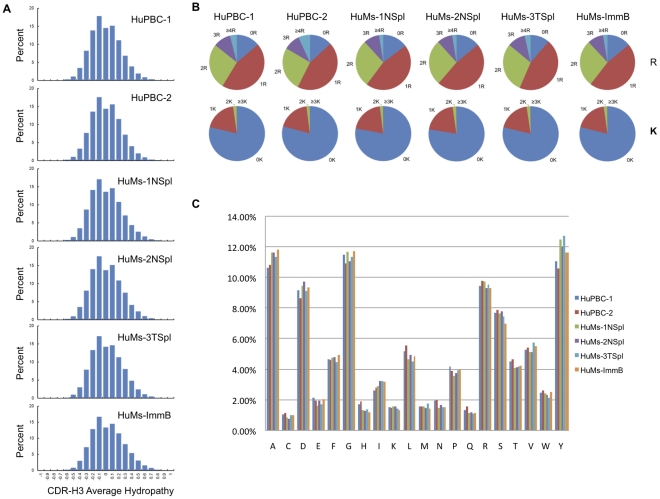
CDR-H3 physicochemical properties imply a normal antibody repertoire in humanized mice devoid of autoimmune signatures. (A) The average hydropathic index of the CDR-H3 interval was calculated using a normalized Kyte-Doolittle scale [38]. The average hydropathy for all samples was centered about the neutral, slightly hydrophilic zero value and all were depleted of either highly charged (≤–0.7) or highly hydrophobic (≥0.7) CDR-H3s. (B) Pie charts showing the statistically similar proportion CDR-H3s with ≥0,1,2,3, or 4 positive charges, namely, the cationic residues arginine (R, *top row*) and lysine (K, *bottom row*). (C) Similarity of individual a.a. distributions within CDR-H3.

The conservation of hydropathy, resulting in selection for a neutral CDR-H3 and against a hydropathically extreme (charged or hydrophobic) antibody-binding site, is a common theme across vertebrate evolution [37]. The quantitative determination of CDR-H3 average hydropathy (based on Kyte-Doolittle scaling [38]) was neutral without a bias towards charged or hydrophobic a.a. usage, yielding a normal distribution centered about the neutral zero value across all HuMs and HuPBC samples (Figure 6A). The frequency of occurrence of charged a.a.s (R or K) within the CDR-H3 interval was also statistically similar between HuMs and HuPBC data sets (Figure 6B). Enrichment for V_H_–proximal R residues in HuMs CDR-H3s constitutes evidence for IgH receptor editing by V_H_ replacement [33]; however, this was not observed. V_H_ replacement can also result in increased CDR-H3 length due to retention of a portion of the 3′ end of the original V_H_ segment [33], and since no change in length was observed within the CDR-H3 primary repertoire this also suggests that V_H_ replacement was not extensive nor somehow aberrant in HuMs repertoires. Although total counts of single R and K residues was uninformative (Figure 6B), a deeper search for highly charged CDR-H3 polymeric motifs (e.g., RRKR) in the data sets was performed in an effort to discern potential autoimmune signatures present at very low abundance; rare (<0.05%) polymeric motifs were observed in all data sets with the same trends (data not shown). These CDR-H3 sequences suggest that a very small population of presumably non-pathogenic, yet autoreactive antibodies may exist in healthy individuals (as well as in HuMs).

**Figure 7:**
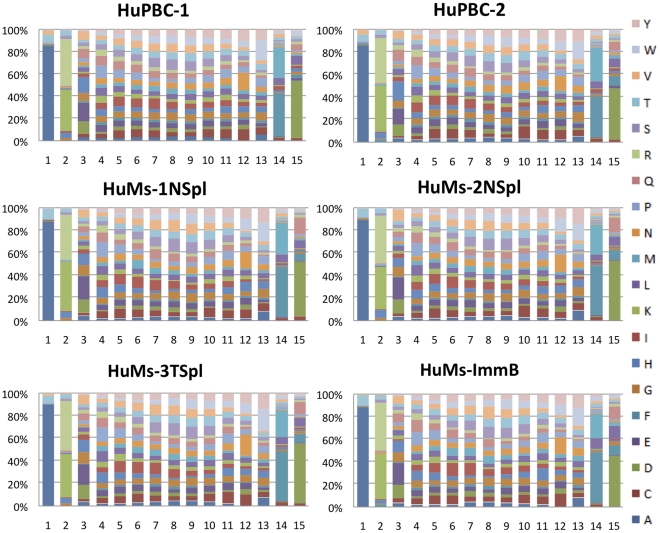
CDR-H3 amino acid patterning. Positional amino acid frequencies in CDR-H3s of the same length. Length n = 15 was the approximate mean length of CDR-H3 observed in all data sets. Positional biases are generally preserved in all sets.

Lastly, CDR-H3 a.a. compositions were determined to display a statistically similar pattern in both HuMs and HuPBC repertoires overall, as the expected prevalence of tyrosine, serine, and glycine was observed, and no distortions of other residues were discerned (Figure 6C). The positional distribution of a.a. abundances within CDR-H3s of a fixed length (e.g., 15 a.a.) was also quantitatively similar (Figure 7). We conclude that the global a.a. content and physicochemical nature of the CDR-H3 repertoire in humanized NOD-*scid*-*IL2Rγ^null^* mice preserve essential evolutionarily conserved features.

### CDR-H3 Repertoires are Extremely Diverse yet are Enriched for Nonrandom, Preferential IGH D-J and V-J Pairings

**Figure 8:**
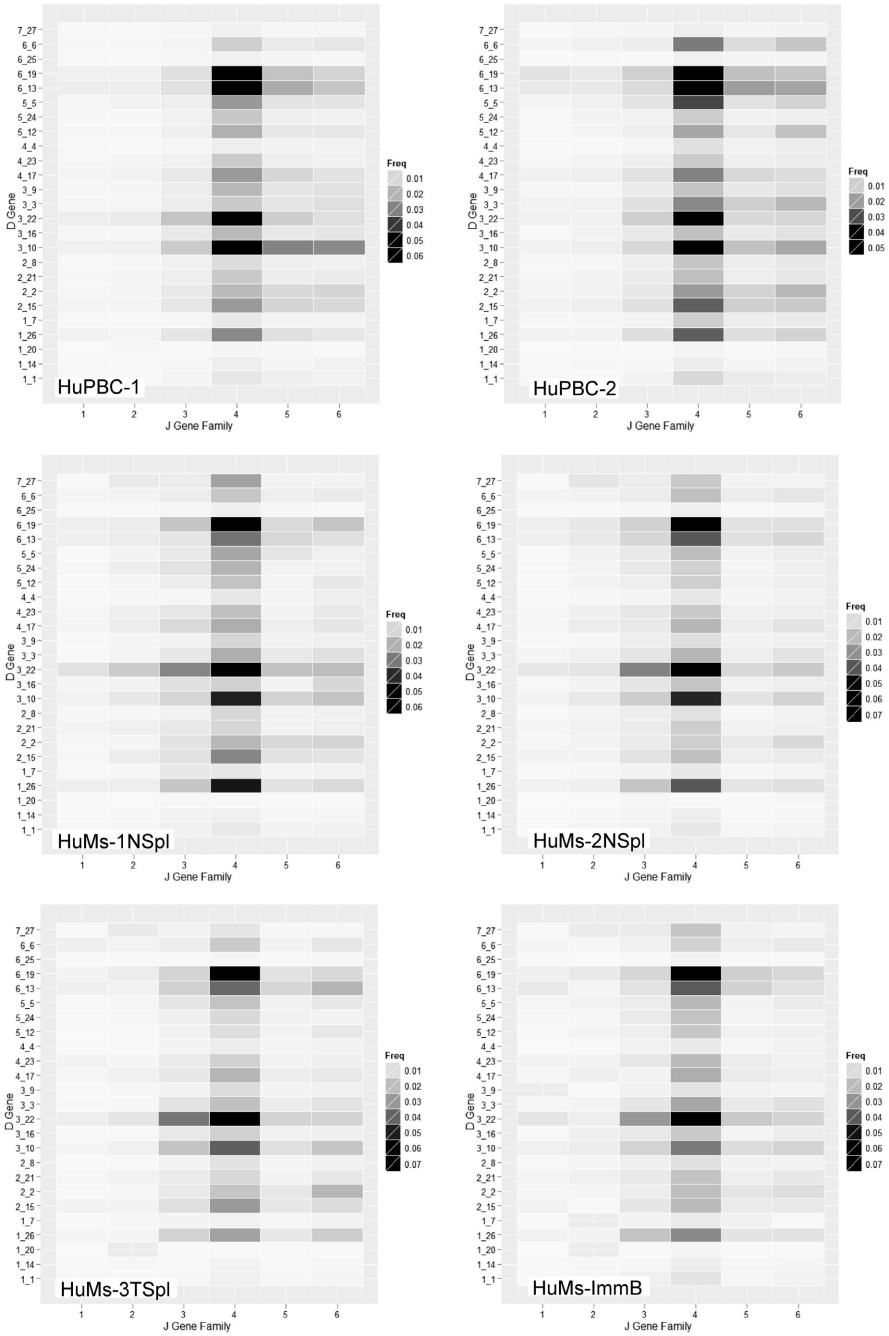
Pairwise preferential D-J joins are observed in both humanized mice and human peripheral B cell immunoglobulin repertoires. Heatmaps of D-J rearrangements for IgM transcripts are shown. Several pairwise associations are overrepresented (*D6-19* with *J4*, *D3-22* with *J4*, and *D3-22* with *J3*) and at comparable frequencies in all of the repertoires. Nonrandom patterns of D-J rearrangement established in the primary repertoire of newly generated IgM^+^IgD^–^ immature B cells of humanized bone marrow (HuMs-ImmB) are chiefly preserved among peripheral humanized B cells (HuMs) which closely resemble those patterns seen in the peripheral blood human B cells (HuPBC).

CDR-H3 is positioned at the center of the receptor binding site and is a principal determinant of Ig specificity [39], [40], [41]. Since the D_H_ gene composes the core of CDR-H3, global biases in D_H_ usage or biases in D-J associations should in turn bias the CDR-H3 repertoire. While CDR-H3 repertoires of HuMs and HuPBC were extremely diverse and unique in a.a. sequence (as above), we nevertheless observed apparently nonrandom preferential D-J rearrangements which were strikingly similar between HuPBC and HuMs (Figure 8). *D6-19* and *D3-22* paired preferentially and abundantly with the *J4* segment at an average of ∼5% and ∼4.5% in HuPBC, and ∼5.5% and ∼7% in all HuMs repertoires, collectively. Overrepresented D-J recombinants have also been observed in another 454 deep-sequencing data set [42]; there, the preferential pairwise recombination of *D3-22* with *J3* was observed, and this recombinant is overrepresented in our HuPBC data sets as well (wherein the frequency of the D-J recombinant is greater than the product of the individual D and J frequencies). Shared preferential V-J segment combinations for V_H_ genes were also noted in the HuMs and HuPBC repertoires (data not shown). *IGHV3-23* and *IGHV3-30* paired with *IGHJ4*, as a particular example, predominated heavily in our cDNA HuPBC-1 and HuPBC-2 data sets (average 5.5%), just as it did in the genomic DNA data set of Boyd *et al.* [42] [average 3.4%], and as it does in the cDNA HuMs repertoires collectively (average 4.8%). Preferential D-J and V-J recombinants challenge the idea that the generation of the primary repertoire is simply random and moreover suggests the possibility that the generation of CDR-H3 diversity is in fact restricted.

### Somatic Mutation in Humanized Mice and Human Peripheral Blood B Cells

The average rate of SHM (mutations per 100 nt) was approximately 5-fold more in HuPBC transcripts (9.8 nt for IgG; 1.7 nt for IgM) than that observed in HuMs (2.3 nt for IgG; 0.3 nt for IgM). The presence of class-switched IgG cells in humanized NOD-*scid*-*IL2Rγ^null^* mice, although under-mutated compared to HuPBC control, nevertheless indicates that the murine stromal milieu permits the spontaneous generation of IgG B cells.

**Figure 9:**
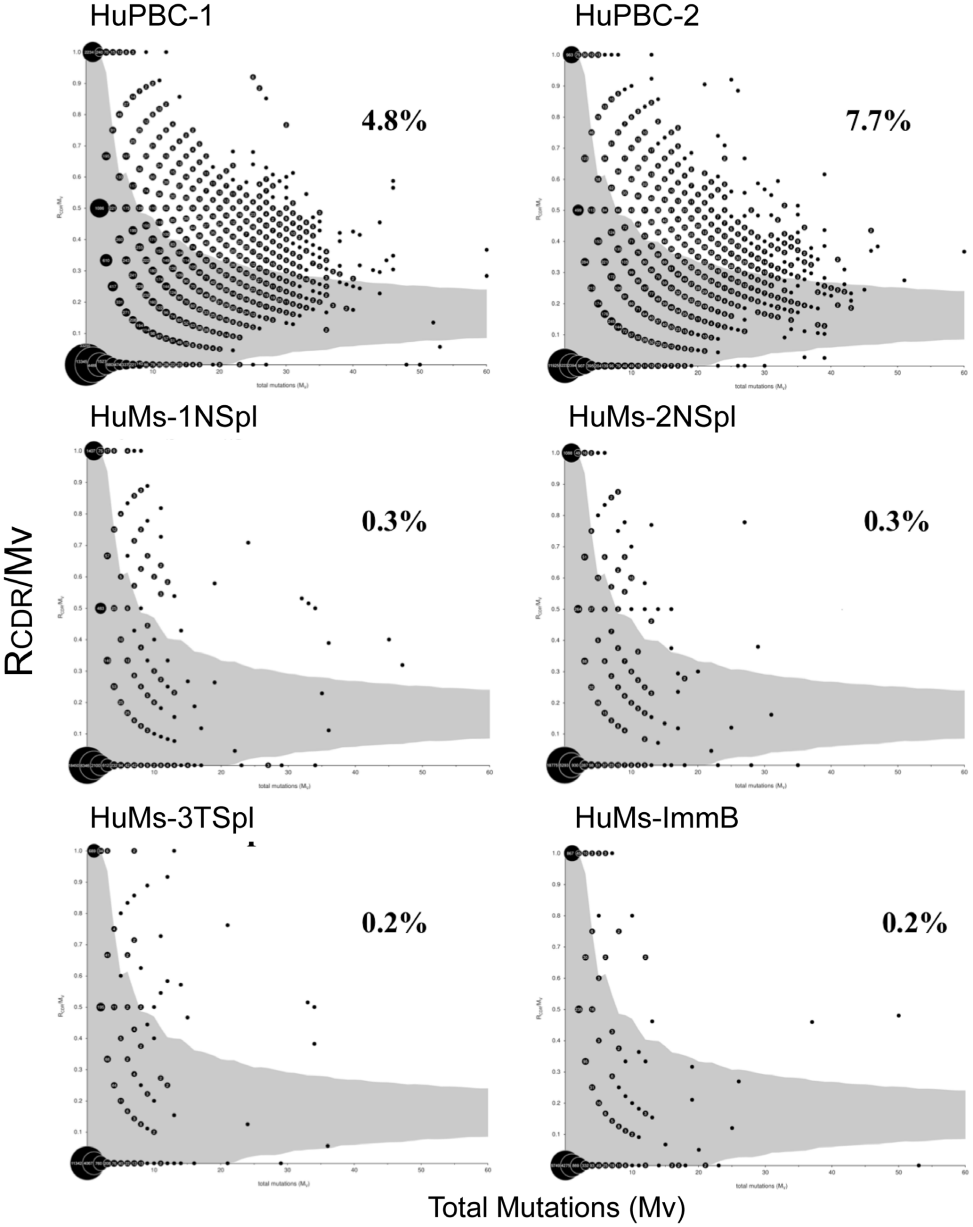
Somatic mutation in IgM primary repertoires. The ratio of replacement mutations in CDR-H1 and CDR-H2 (*R*_CDR_) to the total number of mutations (*M*_v_) is plotted on the *y*-axis versus *M*_v_ on the *x*-axis. The 95% confidence limit for the probability of random mutations is shaded in gray. Data points falling outside this limit represent IgM sequences with a significant occurrence of replacement mutations in the CDRs and are considered indicative of antigen selection. Data points are numbered according to their observed frequency. The overall paucity of somatic mutation in the V_H_ gene among humanized B cells is visually apparent. Replacement mutations in CDR-H1 or CDR-H2 are rare. Whereas this result is expected for humanized samples HuMs-1 and HuMs-2 (naïve CD19^+^IgD^+^CD27^–^ splenic B cells), and expected for HuMs-ImmB (newly formed, immature bone marrow B cells), HuMs-3 was prepared from bulk spleen and could conceivably have captured a subpopulation of somatically mutated human IgM^+^ memory cells. Whereas greater than 99% of humanized HuMs B cells show no evidence for antigen selection (nonrandom mutation), HuPBC cells show an approximately ten-fold (4.83%) to twenty-fold (7.65%) greater proportion of antigen-selected sequences.

The ratio of replacement mutations in CDR-H1 and CDR-H2 (*R*_CDR_) to the number of total mutations (*M*_v_) in the V_H_ gene among IgM transcripts was also calculated (Figure 9). An average of <0.3% HuMs IgM transcripts showed indications of antigen selection. Even though HuMs-3TSpl potentially contained a subpopulation of mutated IgM^+^ memory cells, since it was derived from total spleen, its composition of antigen-selected sequences was also ∼0.3%, nearly identical to the sorted naïve cells in HuMs-1NSpl and HuMs-2NSpl. HuPBCs, by contrast, showed an average of 6.2% antigen-selected sequences, which is >20 fold greater than in HuMs and reflects the presence of somatically mutated IgM^+^ memory cells and plasmablasts found normally in PBMC. Collectively, our results show that somatic selective forces potentially exerted by the murine stroma fail to enrich for CDR-H1 or CDR-H2 a.a. replacements.

## Discussion

The humanized mouse model has proven to be invaluable for biomedical research to address a variety of fields such as hematopoiesis, the study of infectious diseases, and cancer [3], [4], [6]. In particular, NOD-*scid*-*IL2Rγ^null^* engrafted mice have been shown to have reasonable levels of T and B cell repopulation and can mount T-cell dependent responses. However, antigen-specific B cell responses in this model are generally poor, with only low titers (<1∶500) emerging in a small percentage (typically 10%) of immunized animals following multiple boosts with strong adjuvants [15], [16] or following administration of B-cell differentiation factors such as BLyS, IL-6, IL-21, or TNF-α. We explored whether developmental defects in the Ig gene repertoire might be partly responsible for the low level of antibody responses in this model. Earlier, Capra and co-workers used single-cell PCR to amplify and sequence a few hundred V_H_ genes from B cells isolated from a somewhat less efficient HuMs model (engrafted NOD/SCID and NOD/SCID/β_2_m^-/-^). Their results indicated that at least at this moderate level of resolution, V_H_ family utilization and CDR-H3 lengths in HuMs resembled those of the human repertoire [25], [26]. The emergence of next-generation high-throughput DNA sequencing platforms and bioinformatics algorithms has now made it possible to sequence and analyze Ig repertoires at an unprecedented level of detail [22], [28], [42], [43], [44].

Our analysis of over 315,000 functional sequences from immature B cells, naïve B cells and total splenocytes now firmly establishes that engraftment of human hematopoietic stem cells into mice yields extensively diverse B-cell repertoires expressing a full suite of V_H_ and V_L_ Ig genes. Combinatorial Ig gene rearrangement in the naïve B cells in humanized mice was indistinguishable from the human peripheral blood B-cell repertoire. The pattern of germline family usage in our PBMC set is in line with recently reported smaller sets of Ig repertoires [29], [31], [45] and thus should provide a useful reference set for future repertoire studies. Moreover, our human data set, unlike most studies, includes repertoire profiling not only of the IgH but also the IgL immune loci.

It is intriguing that the highly correlated relationship to the mouse-derived human B-cell subsets was not biased by antigen-experienced cells in the human PBMC samples (memory [that can exceed >50% of the peripheral B cells] and plasma cells). First, to avoid bias from overcounting highly-transcribing cells (such as plasma cells), we counted unique VDJ rearrangements (hence, unique CDR-H3s) only once so as to avoid redundancy in the analysis and to normalize transcript counts in the expression libraries. Using a similar strategy to count only unique VDJ rearrangements, and in line with our observations, the very recent report by Glanville et al. [46] has also confirmed a surprising, highly correlated Ig gene-segment use between memory and naïve B cells among normal human PBMCs. This suggests that the compartment of memory B cells, although shaped by antigen-driven and putatively stochastic selective forces, is sampling repeatedly from a genetically predetermined repertoire of naïve B cells. This provides an insight as to why we observed strong correlation in Ig germline family use in humanized mice and human peripheral blood B cells even though we did not precisely compare subset-for-subset between the two samples.

In all data sets, CDR-H3 repertoires were extremely diverse and unique in a.a. sequence, nearly devoid of overlapping or coincident Ig sequences. We observed CDR-H3 junctional diversity to be highly similar in IgM humanized and human data sets, indicating that all functional attributes of the VDJ enzymatic machinery are completely functional in these mice. Physicochemical properties of the CDR-H3 interval were strictly conserved between humanized and human data sets, too: categorical constraints were observed for overall a.a. composition, average hydropathy, charge distribution, length, and the quintessential enrichment for the critical antigen-recognition residues tyrosine, glycine, and serine. The CDR-H3 interval determines and delimits antibody functionality, as documented in gene-targeted mouse models [47], [48], and insofar as evolutionarily conserved CDR-H3 characteristics are globally preserved in engrafted NOD-*scid*-*IL2Rγ^null^* mice, we conclude that humanized antibody repertoires are quantitatively analogous in diversity, composition, and potential functionality (antigen binding) compared to human peripheral B cells. A corollary directly stemming from this finding is that the repertoire in humanized mice must be shaped by B-cell intrinsic mechanisms or else repertoire-shaping signals for the mouse and human cellular microenvironments [49], [50] are indistinguishable for generation of the primary repertoire at the time point examined in our study. It remains to be clarified whether differences in repertoire-shaping signals might be revealed in other contexts, however, such as during normal aging or following antigen-specific challenge. Additionally, the correlated frequency of preferential pairwise D-J combinations we observed among humanized and human peripheral B-cell repertoires further suggests an intrinsic determination of antibody diversity that is non-random, as suggested by others [42], [48], [51], [52].

Developmental checkpoint regulation has been described for the counterselection of autoantibody CDR-H3 during normal human B-cell development [17]. Autoantibody sequences typically display altered CDR-H3 hydropathy, increased length, and enrichment for charged amino acids [37]. Additionally the *IGHV4-34* and *IGKV4-1* genes are inherently autoreactive and have been implicated in B-cell mediated autoimmune diseases and dysregulated B-cell tolerance [11], [12], [13], [18], [34]. Although these are not the only V genes to have been correlated with autoimmunity [53], they are perhaps the best described and by multiple research groups. As described in elegant studies by Meffre, Nussenzweig and co-workers, autoantibodies are generated early in B-cell development but are deleted at two checkpoints during differentiation to mature B cells [17], [54]. Although our observations stem from a single sample of immature B cells derived from the pooling of three HuMs, the reduction in the usage of the inherently autoreactive *IGHV4-34* gene and the SLE-associated *IGKV4-1* gene in the periphery compared to the newly formed immature B cells presents compelling evidence that such a checkpoint in the regulation of the repertoire is operational in humanized mice. That other key characteristics of this single pool of cells are preserved (e.g., IGHD and IGHJ usage, CDR-H3 character) provides a measure of confidence in sample integrity; furthermore, the repertoire profiling of such a large number of immature B cells from this single pool (55,000 sorted cells) also provides a measure of confidence in the integrity of the sample and minimizes the possibility of statistical skewing due to undersampling. Lastly, there is considerable interest to further “humanize” humanized mouse models by the site-specific knock-in of human cytokine transgenes [55], yet the absence of the pro-survival human cytokine BAFF/BLyS in our model would have been predicted to reveal a greatly diminished frequency of potentially autoreactive B-cell receptors (e.g., IGHV4-34 ) in humanized repertoires compared to human blood B cells, as models have proposed a greater dependency of autoreactive B cells on BAFF/BLyS for continued survival [56]; however, we observed instead a significantly greater frequency of IGHV4-34 in all humanized mouse data sets compared to human blood B cells, suggesting indirectly the existence of peripheral tolerance mechanisms independent of the BAFF/BLyS pathway, in agreement with a recent report [57], although the effect of BAFF/BLyS on human B cells in humanized mice has yet to be directly demonstrated [55].

The developmental regulation of *IGHV4-34* in humanized mice is intriguing since this V_H_ segment invariably encodes for natural autoantibodies reactive for the I/i carbohydrate antigens expressed on erythrocytes (and on other cell types) and is considered intrinsically autoreactive because there is no requirement for somatic mutation of the germline gene to confer this specificity [12], [35]. The unregulated proliferation of *IGHV4-34* B cells can result in pathological cold agglutinin disease characterized by serum antibodies, usually IgM, directed against erythrocytes, implying that tight regulation of *IGHV4-34* B cells must be required to minimize the occurrence of autoimmune disease. Not only does this apply to the long-standing observations in SLE disease mentioned above, but its relevance has been underscored very recently in patients with AID deficiency and hyper-IgM autoimmune syndrome who manifest defective checkpoints in B-cell tolerance as determined by single-cell cloning, repertoire analysis, and recombinant antibody expression [19]; these patients express an abnormal Ig repertoire and significantly increased autoreactive antibodies which are highly enriched for precisely one V gene compared to healthy donors, namely, *IGHV4-34*. In addition to *IGHV4-34* involvement in autoimmunity, a profound counterselection against *IGHV4-34* among normal peripheral B-cell subsets (GC, memory, and PC) has also been documented in healthy subjects [12], [13]. There is evidence to suggest that *IGHV4-34* is selected into an unusual lineage of IgD class-switched peripheral B cells (enriched for the *IGKV4-1* light chain gene, too) which has been proposed to be a “sink” for B cells expressing autoreactive receptors in normal humans [34].

Our analysis did not reveal a counterselection of CDR-H3 autoimmune signatures in the transition from the immature B cell stage in bone marrow to the naïve B-cell stage in the periphery, indicating that deletion of such CDR-H3s occurs earlier during development in the bone marrow of humanized mice. Consistent with this hypothesis, Wardemann *et al.* [17] described a first developmental tolerance checkpoint which occurs during the transition between early IgM^–^ precursors and newly formed IgM^+^ immature B cells. Efforts to determine the early B-cell repertoire in humanized mice were not successful due to the temporal nature of engraftment: at the point where B-cell development has peaked (14–18 weeks post-engraftment) the number of pro-B and pre-B cells that could be isolated from the bone marrow was vanishingly small.

Our collective observations indicate that human hematopoietic progenitors transplanted into NOD-*scid*-*IL2Rγ^null^* mice rearrange Ig genes to generate B cells expressing a diverse primary antibody repertoire enriched for unique CDR-H3s, bearing an extraordinarily high degree of similarity to the natural antibody landscape of normal human peripheral B cells, yet with evidence for the clonal deletion of potentially autoreactive V genes suggesting that normal tolerance mechanisms remain intact in these mice. Extensive repertoire profiling in the age of systems immunology–utilizing high-throughput sequencing followed by rapid analysis through bioinformatics computing–is an emerging area of investigation which should continue to expand a truly global examination of Ig repertoires as well as enhance a deeper understanding of humoral immune responses and assist monoclonal antibody discovery in mice [22] and other species.

## Supporting Information

Figure S1 **Spearman correlation of IGHV gene use between HuMs and HuPBC samples.** Shown is a grid of percent frequency of IGHV gene utilization and a plot of these frequencies as a scatter graph. In each graph, HuPBC samples (IgM+IgG) are plotted against either peripheral HuMs-Spl samples (HuMs-1NSpl, HuMs-2NSpl, HuMs-3TSpl) or the centrally derived HuMs-ImmB sample.
(TIF)

Figure S2 **Spearman correlation of IGKV gene use between HuMs and HuPBC samples.** Shown is a grid of percent frequency of IGKV gene utilization and a plot of these frequencies as a scatter graph. In each graph, HuPBC samples (IgM+IgG) are plotted against either peripheral HuMs-Spl samples (HuMs-1NSpl, HuMs-2NSpl, HuMs-3TSpl) or the centrally derived HuMs-ImmB sample.
(TIF)

Figure S3 **Spearman correlation of IGLV gene use between HuMs and HuPBC samples.** Shown is a grid of percent frequency of IGLV gene utilization and a plot of these frequencies as a scatter graph. In each graph, HuPBC samples (IgM+IgG) are plotted against either peripheral HuMs-Spl samples (HuMs-1NSpl, HuMs-2NSpl, HuMs-3TSpl) or the centrally derived HuMs-ImmB sample.
(TIF)
